# Eaten up by boredom: consuming food to escape awareness of the bored self

**DOI:** 10.3389/fpsyg.2015.00369

**Published:** 2015-04-01

**Authors:** Andrew B. Moynihan, Wijnand A. P. van Tilburg, Eric R. Igou, Arnaud Wisman, Alan E. Donnelly, Jessie B. Mulcaire

**Affiliations:** ^1^SOCO-UL Lab, Department of Psychology, Education & Health Sciences Faculty, University of LimerickLimerick, Ireland; ^2^Centre for Research on Self and Identity, School of Psychology, University of SouthamptonSouthampton, UK; ^3^School of Psychology, Keynes College, University of KentCanterbury, UK; ^4^Centre for Physical Activity and Health Research, Physical Education and Sport Sciences Department, Education & Health Sciences Faculty, University of Limerick,Limerick, Ireland; ^5^School of Medicine, University of St. AndrewsFife, UK

**Keywords:** boredom, self-awareness, individual differences, sensation-seeking, unhealthy eating, meaning

## Abstract

Research indicates that being bored affectively marks an appraised lack of meaning in the present situation and in life. We propose that state boredom increases eating in an attempt to distract from this experience, especially among people high in objective self-awareness. Three studies were conducted to investigate boredom’s effects on eating, both naturally occurring in a diary study and manipulated in two experiments. In Study 1, a week-long diary study showed that state boredom positively predicted calorie, fat, carbohydrate, and protein consumption. In Study 2, a high (vs. low) boredom task increased the desire to snack as opposed to eating something healthy, especially amongst those participants high in objective self-awareness. In addition, Study 3 demonstrated that among people high in objective self-awareness, high (vs. low) boredom increased the consumption of less healthy foods *and* the consumption of more exciting, healthy foods. However, this did not extend to unexciting, healthy food. Collectively, these novel findings signify the role of boredom in predicting maladaptive and adaptive eating behaviors as a function of the need to distant from the experience of boredom. Further, our results suggest that more exciting, healthy food serves as alternative to maladaptive consumption following boredom.

## Introduction

Recent studies have highlighted that general measures of negative affect may not be strongly associated with eating behaviors (Evers et al., 2010; Adriaanse et al., 2011; Haedt-Matt and Keel, 2011). However, specific types of negative affect seem to play a more important role in understanding some food consumption (Goldenberg et al., 2005). One likely, less researched affective predictor of eating behavior is boredom (Koball et al., 2012). Boredom has been implicated in the current obesity epidemic and eating behavior (World Health Organization-Europe, 2007). Eating when bored follows a psychological process that is distinct from those prompted by other negative states in the sense that boredom has been found to predict eating behaviors independently of other negative emotions. Specifically, boredom continues to be an important predictor of eating behaviors after controlling for other affective states (Koball et al., 2012). This suggests that boredom can help to explain some eating behavior (Cleobury and Tapper, 2014). Recent theories suggest that eating in response to boredom may be a meaning-regulation response (Wisman, 2006), unlike what happens regarding some other negative emotions (Van Tilburg and Igou, 2011). However, the literature on boredom and eating has not yet established a causal relationship between state boredom, a relatively recent psychologically examined construct, and eating.

Boredom is a discrete emotion that associates with feeling dissatisfied, restless, and unchallenged when one interprets actions and situations at the present time as purposeless (e.g., Leary et al., 1986; Mikulas and Vodanovich, 1993; Barbalet, 1999; Fahlman et al., 2009; Van Tilburg and Igou, 2011, 2012). When one is bored, situations receive low attention due to lack of interest or stimulation coming from one’s immediate environment (Sansone et al., 1992). Essentially, the emotional experience of boredom signals that a current situation is devoid of purpose (Barbalet, 1999; Van Tilburg and Igou, 2012). Following meaning threats, people are motivated to re-establish a sense of purpose in their engagement or turn to behaviors that they deem more meaningful. Indeed, boredom is a meaning threat (Barbalet, 1999; Van Tilburg and Igou, 2012) and triggers responses aimed at reinstating a sense of purpose. For example, Van Tilburg and Igou (2011) found that boredom increased people’s valuations of their in-groups and devaluation of out-groups, key sources of meaning (Heine et al., 2006). Similarly, people sometimes engage in nostalgic reverie to counteract the lack of meaning signaled by boredom (Van Tilburg et al., 2013). Specifically, boredom serves the self-regulatory function of redirecting people’s cognitions and behaviors toward mitigating the lack of meaning at hand (Van Tilburg and Igou, 2012).

In the context of a compromised sense of meaningfulness, as signaled by boredom, different types of responses, relevant to health behaviors, have been implicated (Goldenberg and Arndt, 2008; Arndt and Goldenberg, 2010). When people face a challenge to perceived meaning, they may attempt to escape from their sense of self-awareness and hence are *distracted* from such threats completely. Therefore, the conflict between the desired sense of meaningfulness and the acute lack of it is removed by losing one’s sense of self-awareness. This is a common, though short-term reaction to lacking perceived meaning. Examples of means to avoid this aversive self-awareness include engaging in unhealthy behaviors that are ultimately harmful. For example, food helps to escape a sense of aversive self-awareness (Wisman, 2006) and may hence be used to cope with boredom. The excitement or stimulation certain foods offer may help to distract people’s attention from the bored self. Indeed, obesity is more prevalent among those who regularly experience boredom, compared with other negative states (Abramson and Stinson, 1977). Consistently, boredom has been correlated with various eating disorders (Ganley, 1989).

Similarly, objective self-awareness theory (e.g., Duval and Wicklund, 1972; Gollwitzer and Wicklund, 1985) suggests that people become more aware of discrepancies between the present and ideal self when the self is the focus of attention. This is a different phenomenon than mindfulness, which involves changing how one becomes aware of one’s thoughts and experiences that are seen as less real, personal, vivid, and compelling, and perceiving them as transient events (Papies et al., 2015). Bored people note the lack of purpose in their current state and how this contrasts from their ideal of engaging in purposeful activities (Van Tilburg and Igou, 2012). This inconsistency is aversive, motivating action to reduce the discrepancy. If people are unable to do so by doing something that gives them a sense of meaning (e.g., bolstering cultural worldviews and ideologies), they become likely to focus on reducing or avoiding the self-focus. Again, one way of avoiding aversive self-related thoughts is by ‘escaping from self-awareness,’ for example, by eating or other behaviors that involve a loss of self-awareness such as affiliation (Heatherton and Baumeister, 1991; Wisman and Koole, 2003; Wisman, 2006). These behaviors shift focus from the self-regulatory challenge to acute sensations.

Thus, we suggest that the state of aversive self-awareness when bored can motivate people to escape the self. If boredom leads to eating, then this effect may in part be understood as an attempt to reduce self-awareness as a means to escape the self-regulatory discrepancy. Further, we reason that people high in dispositional self-awareness, that is, people who gravitate to introspection easily, would be particularly vulnerable to the aversive self-discrepancies. Since eating distracts people from unpleasant self-awareness associated with lacking meaning (Gerrard et al., 2008), people especially high in self-awareness should engage in eating when they experience boredom.

Our current research is designed to go beyond previous research, analyzing food choices people make in relation to state boredom. These predictions were examined in a series of three studies. Study 1 used a diary procedure examining state boredom and food intake. State boredom was manipulated in Studies 2 and 3 and consumption intentions and behavior were measured whilst examining self-awareness as critical moderator. Further, in Study 3, we investigated whether boredom can promote eating more exciting, healthier foods, as more adaptive eating behavior than indulging in less healthy foods. Boredom encourages people to seek sensation (e.g., Van Tilburg and Igou, 2012), therefore, we argue that more ‘exciting’ food serves as a potent distraction of self-regulatory challenges for example by providing an intense appearance or taste (McGregor et al., 2012). Consequently, the current research tested if the negative effects of boredom on eating behaviors would be counteracted by offering more exciting, yet healthy foods. We expected that bored people would eat certain foods as a function of escaping from aversive self-awareness associated with the meaning threat. Indeed, self-awareness is necessary to perceive that one is experiencing meaning threats (Wisman, 2006).

Our three studies adopted different methodological approaches (diary study, experimental) in which eating was assessed differently (self-reported, behavioral). By examining the link between boredom and eating from these different angles, limitations associated with one particular approach were addressed with another methodology. For example, the diary study offered a window in real-life settings which were complemented by test of causal relations available through lab experimentation. All studies were approved by the University of Limerick’s education and health sciences research ethics committee.

## Study 1: A Behavioral Diary Study: Boredom Increases Unhealthy Eating

In this study, we used a 1-week long diary procedure to examine the relationship between state boredom and food intake. We predicted that boredom was positively associated with increased intake of food as indicated by increases in energy, fats, carbohydrates, and proteins.

### Method

#### Participants and Design

Thirty-three people from Limerick city, Ireland (*M*_age_ = 50.30, SD = 10.81; 30 women, 3 men; *M*_BMI_ = 24.96, SD = 5.37, range: 17.07–43.40) took part in a diary study across 7 days. Participants were recruited from the general population by distributing advertisements in the local area.

#### Materials and Procedure

Participants received a folder with the paper-and-pencil materials of the diary study sorted by day. On the first day and after giving informed consent, participants reported demographic information as well as height and weight. Further, they were instructed how and when to complete the diaries each day. Participants then completed a shortened version of the ‘boredom proneness scale’ (Gordon et al., 1997). This scale assessed individual differences in the proclivity to experience boredom (12 items, e.g., “It is easy for me to concentrate on my activities”; 1 = *not at all*, 7 = *very much*; α = 0.66). Trait boredom proneness is psychologically different from state boredom (Van Tilburg and Igou, 2012). This scale was included to verify that state boredom prompted increased eating of certain food groups above and beyond any influence of trait boredom.

This was followed by the ‘Positive and Negative Affect Scale’ short-form (PANAS; Watson et al., 1988), which probed negative and positive affect with five items for each construct (e.g., upset; 1 = *very slightly* or *not at all*, 5 = *extremely*; α_positive_ = 0.70, α_negative_ = 0.71). Participants were asked to answer this scale based on how they felt in general.

On each evening, participants first responded to three separate items developed by the research team assessing state boredom, stress, and enjoyment experienced during that day (e.g., “How boring was your day?,” “Did you have a stressful day?,” “Did you have an enjoyable day?”; 1 = *not at all*, 7 = *a lot*). These items were taken from existing reliable and valid measures to fit the current diary study context (Van Tilburg and Igou, 2011, 2012). Next, participants kept track of their food and drink intake using the 7-days EPIC-Norfolk diary (Bingham et al., 2001). This diary consists of a booklet with separate sections for each day. Participants were requested to be as detailed as possible in their records and the booklets contained color pictures of various food types and amounts to aid the assessment of portions. Studies assessing the amount and types of different food groups and products consumed attest to the validity and reliability of this measure based on correlations with potassium, nitrogen, and sodium in urine secretion content and weighed records (Bingham et al., 1997; McKeown et al., 2001). Listed consumptions were decoded into daily amounts of energy (kilocalories), fat, carbohydrate, fiber, and protein (grams) using the aids to calculation of food composition provided by McGuire and Beerman (2007).

### Results

Considering the multilevel structure of the data (i.e., multiple days nested ‘within’ participants), data was first disaggregated. Conducting this technique resulted in 231 part-dependent observations (i.e., seven observations for each of the 33 participants). With the exception of the food content variables, all variables were standardized. When using regression-based analyses (multiple regression analysis, simple slope analysis, some multilevel analysis), it is important to standardize variables. This prevents multicollinearity when using interaction terms (Tabachnick and Fidell, 2008). Further, the strength of the covariates correlations with each other and with boredom ranged from small to medium strength (Maximum: 0.65, Minimum: -0.60). Therefore, there was no problematic multicollinearity. Subsequent analysis consisted of restricted maximum likelihood random-intercept multilevel analyses in which participants were specified as a grouping variable, hence accounting for dependency of observations. To examine the impact of state boredom on dietary choices, participants’ daily level of boredom was specified as predictor of the various food contents, with covariates added to more complex models.

Variables were entered into the model based on their relevance or interest to the current study. Specifically, state boredom, the central variable of interest, was entered as the predictor in the first models, and then the other predictors were added in the subsequent models.

#### Energy

Energy consumption was operationalized as kilocalorie content. As reflected in **Table 1** (Model 1), state boredom was significantly positively associated with energy consumption, λ = 98.69, *S*_e_ = 35.90, *t*(215.55) = 2.76, *p* < 0.01. This indicated that when participants’ level of boredom rose by its standard deviation, 1.585, the equivalent energy content of one additional scrambled egg or banana, approximately an extra 100 calories, was consumed.

**Table 1:** Daily consumption of food types as a function of state boredom (Study 1).

	State boredom		State enjoyment		State stress		Boredom proneness		Positive affect		Negative affect		BMI			
Energy	λ	*S_e_*	λ	**S*_e_*	λ	**S*_e_*	λ	*S_e_*	λ	*S_e_*	λ	**S*_e_*	λ	**S*_e_*
Model 1	98.64**	35.69												
Model 2	123.78**	39.77	135.87**	44.68	-1.15	43.94	-33.68	90.77	-16.53	87.18	-56.77	85.20	-24.78	77.59
**Fat**
Model 1	5.25**	1.69												
Model 2	6.15**	1.88	4.67*	2.12	1.17	2.08	1.06	4.62	-3.30	4.44	-4.22	4.34	-3.36	3.96
**Carbohydrate**
Model 1	9.81*	4.64												
Model 2	11.10*	5.20	12.32*	5.83	-3.61	5.74	-1.19	11.30	1.73	10.85	-2.01	10.61	-3.22	9.65
**Protein**
Model 1	3.52*	1.78												
Model 2	4.89*	1.99	5.68*	2.23	0.99	2.20	-2.81	4.49	0.56	4.31	-3.16	4.21	1.05	3.83
**Fiber**
Model 1	-0.23	0.59												
Model 2	-0.09	0.66	-0.21	0.52	-0.31	0.44	-0.04	1.66	1.22	1.60	0.52	1.56	-0.93	1.42

*p < 0.05; **p < 0.01.

The association with daily boredom remained after additionally controlling for the other variables, dispositional boredom, positive affect, negative affect, stress and body mass index, λ = 123.78, *S*_e_ = 39.77, *t*(179.19) = 3.11, *p* < 0.01 (Model 2).

#### Fats

In **Table 1** (Model 1), it can be seen that state boredom was significantly positively associated with the consumption of fats, λ = 5.25, *S*_e_ = 1.69, *t*(215.50) = 3.11, *p* < 0.01. This result indicated that, on average, with every standard deviation increase in boredom, approximately five additional grams of fat were consumed, equivalent to the fat content of one biscuit. This relationship was also found after controlling for the other variables, λ = 6.15, *S*_e_ = 1.88, *t*(177.19) = 3.28, *p* < 0.01 (Model 2).

#### Carbohydrates

As shown in **Table 1** (Model 1), state boredom was significantly, positively associated with carbohydrate intake, λ = 9.81, *S*_e_ = 4.64, *t*(215.92) = 2.12, *p* = 0.04. This result indicated that, on average, with every standard deviation increase in boredom, approximately ten additional grams of carbohydrates were consumed, equivalent to the carbohydrate content of a packet of sweets. This association remained after controlling for the other variables, λ = 11.10, *S*_e_ = 5.20, *t*(180.28) = 2.13, *p* = 0.03 (Model 2).

#### Proteins

In **Table 1** (Model 1), it is shown that state boredom was significantly positively associated with the amount of protein consumed, λ = 3.52, *S*_e_ = 1.78, *t*(215.96) = 2.00, *p* = 0.05. This result indicated that, on average, with every standard deviation increase in boredom, approximately three-and-a-half additional grams of protein were consumed, equivalent to the protein content of a cup of mushrooms. Again, the results remained much the same when controlling for the other variables, λ = 4.89, *S*_e_ = 1.99, *t*(179.47) = 2.46, *p* = 0.02 (Model 2).

#### Fibers

In contrast to the other content indexes, state boredom was not significantly associated with the consumption of fiber, λ = -0.23, *S*_e_ = 0.59, *t*(214.04) = 0.39, *p* = 0.70, as shown in **Table 1** (Model 1), a relation that remained non-significant when controlling for the other variables, λ = -0.09, *S_e_* = 0.66, *t*(176.84) = 0.13, *p* = 0.89 (Model 2).

### Discussion

Consistent with our predictions, state boredom was associated with a greater energy intake, as well as the consumption of higher quantities of fats, carbohydrates, and proteins. Each of these associations remained significant after controlling for stress, enjoyment, and individual differences in boredom proneness, positive affect, negative affect, and body mass index. Also, the impact of these individual differences was not significant.

These findings demonstrate that state boredom indeed relates to higher levels of consumption including fats and carbohydrates, energy-dense food groups most relevant to the obesity epidemic (World Health Organization-Europe, 2007). From our review of the literature, we suspect that this is the first study which has demonstrated overconsumption of particular food groups in response to state boredom.

## Study 2: The Role of Self-Awareness in Boredom and Snacking Desire

A major benefit of Study 1 was that it examined boredom’s relationship with eating behaviors in ‘real-life’ settings. However, the diary method limited testing the causal structure of this relationship. Therefore, our next study tested the causal relationship between boredom and eating behavior in a more controlled context. We designed Study 2 to manipulate boredom and to test the proposed effect of boredom on food preferences at different levels of objective self-awareness. Our hypothesis was that unhealthy eating would function as an escape from aversive self-awareness associated with boredom (Wisman, 2006). Earlier research has suggested that self-awareness required for self-regulation after meaning threats may be compromised as it highlights deficiencies in the self (Twenge et al., 2003; Baumeister et al., 2005). Further, objective self-awareness is an individual difference (Baumeister, 1991). Accordingly, we expected that especially among those participants high on trait levels of objective self-awareness, high levels of boredom would facilitate temptations to snack on less healthy foods compared to those low in self-awareness and those in the low boredom condition.

Self-report measures were used to assess these food choices. By using short self-report measures (rather than elaborate diaries or actual eating behavior), Study 2’s hypothesis was tested in a larger sample, not hindered by more demanding methodological requirements, to control demand characteristics and item redundancy.

### Method

#### Participants and Design

Seventy-nine students (*M*_age_ = 19.68, SD = 2.32; 33 women, 46 men) of the University of Limerick, Ireland participated in Study 2 in return for partial course credits or € 3. They were assigned to one of two conditions of a between subjects design (boredom: high vs. low). This study’s population was changed from Study 1. We did this to generalize findings to other populations (Larson and Richards, 1991).

#### Pilot Study

Importantly, whereas prior studies have manipulated boredom by varying the duration of involvement in a dull activity (e.g., Van Tilburg and Igou, 2011; Van Tilburg et al., 2013), we attempted to keep the task duration comparable across the two studies. For this purpose, a novel boredom induction task was used, based on a pilot study. This task consisted of a simple puzzle in which participants had to connect different objects while adhering to basic rules. In the low boredom condition, several depicted cows and chickens needed to be connected, by drawing a line, to a trough or coop, respectively. Drawn ‘paths’ were not to cross, there was a limit to the amount of animals connected to each trough or coop, and several dotted lines called ‘canals’ could not be crossed. In the high boredom condition, the puzzle was identical except that the cows, chickens, troughs, and coops were replaced with circles, rectangles, triangles, and squares, respectively. Based on past research, we anticipated the latter variation to be less engaging, and therefore to solicit boredom (Csikszentmihalyi, 1975/2000; Van Tilburg and Igou, 2012).

Fifteen students from the University of Limerick worked on both puzzles and evaluated task boredom (“How boring is this task?”; 1 = *not at all*, 7 = *very much*, Van Tilburg and Igou, 2012). The high boredom version was considered more boring (*M* = 5.33, SD = 1.18) than the low boredom version (*M* = 3.80, SD = 1.97), *t*(14) = 3.44, *p* < 0.01, *d* = 0.94.

#### Materials and Procedure

After giving informed consent and providing demographic details, participants completed the eight-item objective self-awareness measure from the self-consciousness scale (Fenigstein et al., 1975; e.g., “I generally pay attention to my inner feelings”; 1 = *not at all*, 7 = *very much*; α = 0.74). This measure was followed by the boredom induction task.

Previous research shows that snacking is associated with less nutritious food (Adriaanse et al., 2011). In addition, people generally try to abide by a societal norm to act healthily, which may be hampered by snacking (Goldenberg et al., 2005). Therefore, as a measurement of unhealthy consumption, participants indicated their desire to snack after the puzzle (“Do you feel like snacking right now?”; 1 = *not at all*, 7 = *very much*) as well as their wish to eat healthy (“Do you feel like eating something healthy right now?,” 1 = *not at all*, 7 = *very much*). These two items were developed by the research team. Afterward, participants were thanked and debriefed.

### Results and Discussion

After standardizing the aggregated objective self-awareness scores and effect coding the boredom induction (-1 = *low*, 1 = *high*), these variables and their interaction were entered as predictors of participants’ reported snacking desires in an ordinary least squares regression analysis. The results of this analysis evidenced the predicted significant interaction between the boredom induction and objective self-awareness, *B* = 0.48, *S*_e_ = 0.24, *t*(75) = 2.00, *p* = 0.05, as well as a significant partial effect of self-awareness, *B* = 0.62, *S*_e_ = 0.24, *t*(75) = 2.59, *p* = 0.01, and a non-significant partial effect of the boredom induction, *B* = 0.22, *S_e_* =0.23, *t*(75) = 0.96, *p* = 0.34.

**FIGURE 1:**
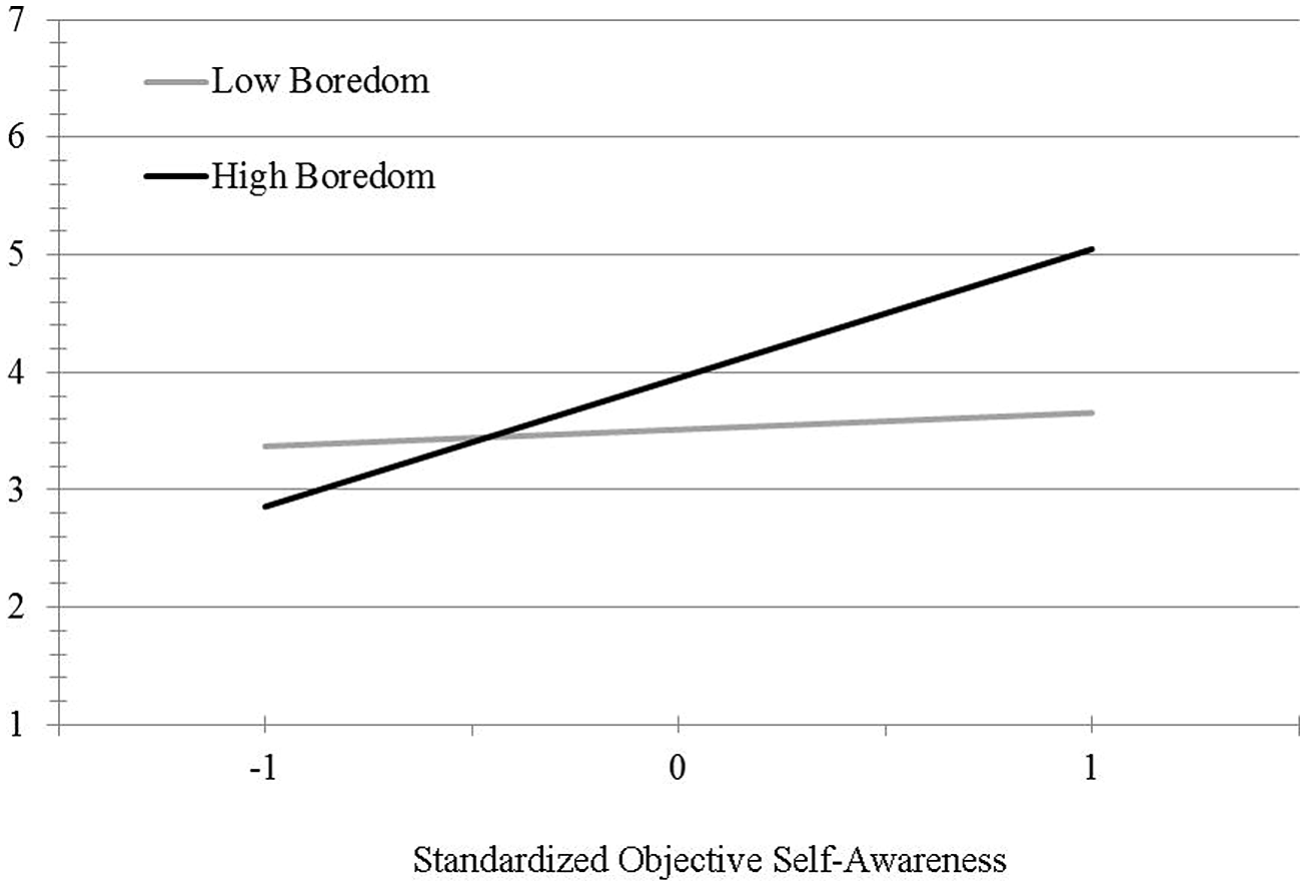
**Snacking desire by boredom and objective self-awareness (Study 2)**.

As illustrated in **Figure 1**, these results indicate that the amplification of snacking desire under high boredom is more pronounced among those high in objective self-awareness relative to those low in objective self-awareness. When probing the interaction (Hayes, 2012, Model 1), no significant association between objective self-awareness and snacking desire was found among those in the low boredom condition, *B* = 0.14, *S_e_* = 0.29, *t*(75) = 0.49, *p* = 0.62. However, there was a significant positive relationship between these two constructs in the high boredom condition, *B* = 1.10, *S_e_* = 0.38, *t*(75) = 2.88, *p* < 0.01. These results indicate that boredom fosters the desire to snack, especially among those high in objective self-awareness.

The results of the healthy eating analysis evidenced no significant interaction between the boredom induction and objective self-awareness, *B* = -0.12, *S_e_* = 0.20, *t*(75) = 0.61, *p* = 0.55, no significant partial effect of self-awareness, *B* = 0.07, *S_e_* = 0.20, *t*(75) = 0.34, *p* = 0.74, and also no significant partial effect of the boredom induction, *B* = 0.19, *S_e_* = 0.19, *t*(75) = 1.02, *p* = 0.31.

Overall, Study 2 demonstrated that state boredom promotes a desire to snack, typically less healthy foods (Adriaanse et al., 2011). The maladaptive eating tendency was especially present among those participants high on objective self-awareness. This finding is consistent with the hypothesis that boredom—which emotionally signals a lack of meaning (Van Tilburg and Igou, 2011, 2012; Van Tilburg et al., 2013)—increases the desire to eat unhealthily, especially among people with high objective self-awareness. Moreover, no effects were observed for the desire to eat healthier foods, suggesting that boredom does not simply increase any consumption, but it promotes more unhealthy consumption in particular.

## Study 3: Boredom Specificity and the Choice of Eating Healthy Foods

In Study 3, we extended the previous studies’ findings by offering participants actual food choices. Further, offering food choices helped to clarify if self-awareness moderates actually acting upon the desire for snacking rather than mere awareness of a desire for snacking. Moreover, boredom specificity was tested against another negative experience, sadness, to rule out that the effects on eating behavior were based on general negative affect repair motivations. Indeed, boredom and sadness have been found to have distinct relationships with eating in previous research (Koball et al., 2012).

We gave participants the choice not only between a healthy vs. less healthy food option, but also a more ‘exciting’ healthy alternative. This was done because seeking stimulation is a distinctive aspect of boredom (Van Tilburg and Igou, 2012). The reason why unhealthy eating is used by bored people to escape from self-awareness may stem from the fact that particularly unhealthy food tends to be more exciting and stimulating. In contrast, healthier food is typically more boring (Craeynest et al., 2008). Importantly, if this excitement factor indeed provides unhealthy food its ego-escape utility, then also relatively exciting alternatives that are nonetheless *healthy* should appeal to those who are bored.

We predicted that among those high in objective self-awareness, boredom would solicit preferences for the less healthy snack. In addition, we expected that the more exciting, healthy alternative would similarly be preferred among bored participants high in objective self-awareness.

### Method

#### Participants and Design

Forty-four students (*M*_age_ = 20.48, SD = 3.12; 31 women, 13 men) of the University of Limerick, Ireland were assigned to one of the two conditions of a between subjects design (boredom vs. sadness) in exchange for € 4.

#### Pilot Study

In a pilot test, fourteen students from the University of Limerick indicated the excitement of the foods using one item (e.g., “These [crackers] are exciting”; 1 = *not at all*, 7 = *very much*). Both the unhealthy sweets (*M* = 5.07, SD = 1.90) and the relatively healthy cherry tomatoes (*M* = 4.07, SD = 1.98) were considered to be more exciting food compared to the relatively healthy crackers (*M* = 2.36, SD = 1.34), *t*(13) = 5.59, *p* < 0.001, *d* = 1.65, and t(13) = 2.43, *p* = 0.03, *d* = 1.01, respectively. The tomatoes and sweets did not significantly differ in perceived levels of their excitement, *t*(13) = 1.36, *p* = 0.20, *d* = 0.52. Although this latter effect size was not trivial, it was much smaller than the difference in excitement judgments between crackers and sweets or tomatoes (*d* = 1.65, *d* = 1.01, respectively). Further, previous literature suggests that tomatoes and sweets have more exciting properties than crackers (Craeynest et al., 2008). Previous research also suggests that tomatoes and crackers are perceived as healthier than sweets (Verhoeven et al., 2012). Thus, participants in the main study had a choice of foods that varied in their healthiness as well as how exciting they were considered to be.

#### Materials and Procedure

After giving informed consent and providing demographic details, participants were seated in a research laboratory and worked on a computer-based study. They first completed the objective self-awareness scale (Fenigstein et al., 1975) as in Study 2 (α = 0.72). Next, participants watched a 15 min movie extract. In the high boredom condition, this movie consisted of an instruction how to set up a successful ‘fish farming’ enterprise, covering themes ranging from selecting suitable species and cages, to water quality and existing aqua culturists’ entrepreneurial insights. The sadness condition also covered water life, but focused on the topic of dolphin abuse specifically. Prior to watching the movie clip, participants were instructed that they could eat as many or as few of the food provided as wanted during the course of the movie. Each participant received three bowls containing 10 cherry tomatoes, sweets, and crackers, respectively. None of the participants indicated to have allergies to any of these products or their content when prompted. All food consumption in Study 3 was measured by counting the remaining snacks following the experiment to assess the amount consumed for each food type.

When the movie clip ended, participants subsequently rated how boring and sad the movie was (e.g., “How boring was this movie?”; 1 = *not at all*, 7 = *very much*), and how bored and sad they felt (e.g., “How bored do you feel?” 1 = *not at all*, 7 = *very much*, Van Tilburg and Igou, 2012). Afterward, participants reported demographics and were thanked, debriefed, and rewarded.

### Results and Discussion

Relative to participants who watched a sad movie, those who watched the boring video clip considered the movie itself as more boring (*M* = 5.00, SD = 1.95, vs. *M* = 1.57, SD = 1.04), *F*(1,42) = 54.58, *p* < 0.001, η^2^ = 0.57, and also felt more bored (*M* = 5.10, SD = 1.84 vs. *M* = 1.65, SD = 0.94), *F*(1,42) = 62.80, *p* < 0.001, η^2^ = 0.60.

#### Unhealthy Food: Sweets

**FIGURE 2:**
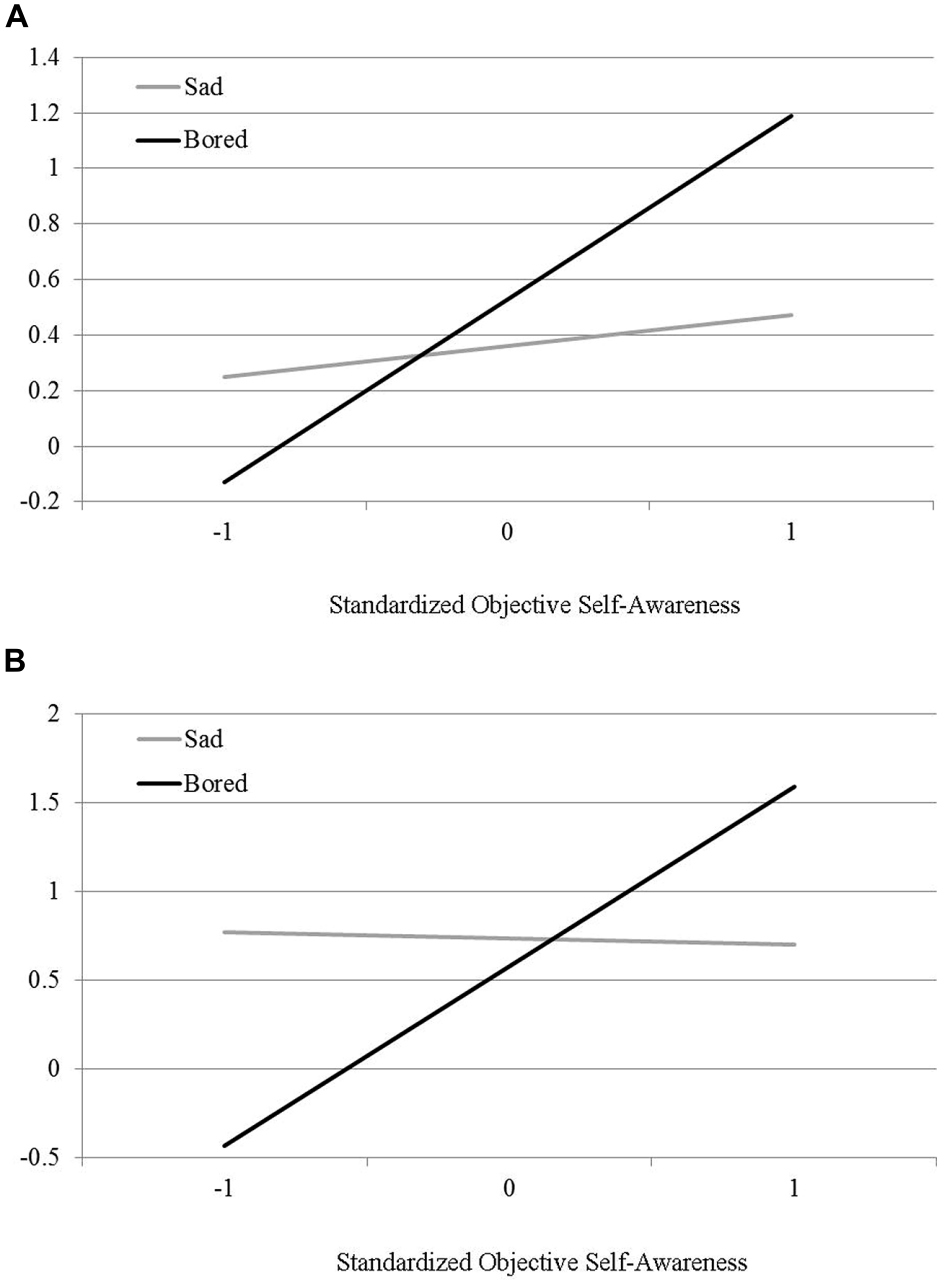
**(A)** Sweets consumption by boredom and objective self-awareness (Study 3). **(B)** Tomato consumption by boredom and objective self-awareness (Study 3).

After standardizing the aggregated objective self-awareness scores and effect coding the movie manipulation (-1 = sad, 1 = boring), these variables and their interaction were entered as predictors of participants’ consumption of the unhealthy sweets in an ordinary least squares regression analysis. The results of this analysis evidenced the predicted significant interaction between the boredom induction and objective self-awareness, *B* = 0.27, *S*_e_ = 0.11, *t*(40) = 2.50, *p* = 0.02, as well as a significant partial effect of self-awareness, *B* = 0.39, *S*_e_ = 0.11, *t*(40) = 3.52, *p* < 0.01, and a non-significant partial effect of the boredom induction, *B* = 0.22, *S_e_* = 0.23, *t*(75) = 0.96, *p* = 0.34. Probing the interaction (Hayes, 2012, Model 1) revealed that objective self-awareness was associated with more consumed sweets among those who were exposed to the boring movie clip, *B* = 0.66, *S*_e_ = 0.18, *t*(40) = 3.67, *p* < 0.001. No significant association was present among those who watched the sad movie, *B* = 0.11, *S_e_* = 0.13, *t*(40) = 0.89, *p* = 0.38, (**Figure 2A**). Thus, the engagement in a highly boring activity promoted the consumption of relatively unhealthy sweets, among those participants high in self-awareness.

#### Healthy Food: Crackers

An analysis similar to the above for the consumption of crackers, the healthy yet unexciting food revealed no significant interaction between the boredom induction and objective self-awareness, *B* = 0.08, *S_e_* = 0.27, *t*(40) = 0.31, *p* = 0.76, no significant partial effect of self-awareness, *B* = 0.08, *S_e_* = 0.27, *t*(40) = 0.29, *p* = 0.77, and a non-significant partial effect of the boredom induction, *B* = 0.02, *S_e_* = 0.25, *t*(40) = 0.08, *p* = 0.94. Thus, boredom did not promote consumption of the unexciting, healthy food as was the case for those who watched the sad movie, *B* = -0.00, *Se* = 0.31, *t*(40) = 0.02, *p* = 0.99.

#### Healthy Food: Cherry tomatoes

A similar analysis was conducted on the consumed cherry tomatoes, a healthy and comparatively more ‘exciting’ food. Intriguingly, a significant interaction between the boredom induction and objective self-awareness emerged, *B* = 0.52, *S_e_* = 0.22, *t*(40) = 2.36, *p* = 0.02, as well as a significant partial effect of self-awareness, *B* = 0.49, *S_e_* = 0.22, *t*(40) = 2.20, *p* = 0.03, and a non-significant partial effect of the boredom induction *B* = -0.08, *S_e_* = 0.21, *t*(40) = 0.39, *p* = 0.70 (**Figure 2B**). Probing the interaction (Hayes, 2012, Model 1) revealed that, objective self-awareness was associated with increased consumption of cherry tomatoes among those who were exposed to the boring movie clip, *B* = 1.01, *Se* = 0.36, *t*(40) = 2.78, *p* < 0.01. No significant association was present among those who watched the sad movie, *B* = -0.03, *S_e_* = 0.25, *t*(40) = 0.13, *p* = 0.89.

## Discussion

Study 3 indicated that boredom promotes preferences for less healthy foods, consistent with the previous studies. As in Study 2, this effect was more pronounced among participants high in self-awareness. Importantly, however, boredom did not just evoke unhealthy eating. Indeed, healthy, yet more exciting foods were also consumed more among high objectively self-aware participants. To our knowledge, these findings have not been identified in any previous research. We acknowledge that watching a movie in a lab with accompanying food probably made participants aware that food consumption was being investigated. However, participants did not know that we related this to boredom (they were unaware of the existence of two conditions) and we assessed emotional experiences after the consumption behavior, essentially ruling out any demand characteristics. Thus, it could be argued that while some participants may have realized that the studies investigated consumption behavior, they are unlikely to have linked this to boredom in particular. These results support the hypothesis that food choices based on boredom are a function of peoples’ efforts to escape from self-awareness.

## General Discussion

Study 1 showed significant, positive relations between state boredom and calorie, fat, carbohydrate, and protein consumption. These relations remained after controlling for stress, enjoyment, and individual differences (e.g., boredom proneness, positive and negative affect, body mass index).

In Study 2, we employed an experimental design and consolidated Study 1 by examining choices between desires to snack on healthy and less healthy food when bored. A boredom manipulation increased the desire to snack, especially amongst those high in objective self-awareness. No such role of objective self-awareness emerged for eating healthily.

Study 3 further extended the findings by investigating boredom’s effects on healthy and more exciting eating choices. In addition, one of boredom’s unique consequences, desire for sensation-seeking, was investigated (Van Tilburg and Igou, 2012). The boredom manipulation increased snacking on sweets and on healthy, more exciting foods (cherry tomatoes), especially among participants high in objective self-awareness. No such interaction emerged for eating healthy, yet unexciting foods. Further, analyses revealed that significant effects were attributed to boredom and no results emerged from sadness (Van Tilburg and Igou, 2011, 2012). Therefore, Study 3 demonstrates that boredom specifically encourages consumption of sensational foods (see also Sommers and Vodanovich, 2000), healthy or unhealthy.

Overall, the empirical findings were derived across a variety of methods and measures, providing convergent support for our hypothesis. For example, the major benefit of using single-item self-report measures in Study 2 comes with the limitation of short self-report measures as main dependent variable. This limitation, however, is in turn addressed by Studies 1 and 3. Collectively, the studies indicate that boredom increases eating, specifically unhealthy and exciting foods which can serve as means to escape the bored self.

Our research supports and extends Wisman’s (2006) theory of eating to escape aversive self-awareness to the domain of boredom. This is particularly relevant for those with high levels of self-awareness. By eating, bored people may regulate their self-awareness to avoid threatening existential issues. Attention is narrowed to the current and immediate stimulus environment (Heatherton et al., 1991; O’Connor et al., 2008). This consumption reduces self-awareness in which the meaning-threat posed by boredom resides.

The key role of escape from self-awareness following boredom (a meaning threat) is what we believe distinguishes this process from solely self-regulatory failure (Vohs and Faber, 2007) and escape-control strategies formulated in earlier research (often trait-like phenomena, relevant to general stress research; Folkman et al., 1986). Previous research has suggested that the capacity for self-control remains largely intact after experiencing meaning-threats (Baumeister et al., 2005). However, an effort to escape from aversive self-awareness and further impulsiveness associated with boredom (Dahlen et al., 2004) are likely to reduce self-regulatory efforts. Thus, the process between boredom and eating behaviors does not seem to be primarily due to a breakdown in self-regulation but a voluntary, active escape from aversive self-awareness.

It could be proposed that escape from self-awareness by unhealthy eating is actually an attempt at hedonic mood repair (Haedt-Matt and Keel, 2011; Loxton et al., 2011). Yet, research strongly suggests that escape from self-awareness is a distinct process. For example, the meaning-regulation strategies employed following boredom are independent of negative affect (Van Tilburg and Igou, 2011, 2012, 2013). Indeed, the predicted effects were obtained by controlling for negative affect (see Studies 1 and 3). Thus, it is unlikely that unhealthy eating as a function of boredom is solely driven by hedonic affect regulation.

Furthermore, it has been reported that people experiencing existential threats become temporarily numb to emotional pain (DeWall and Baumeister, 2006; Twenge et al., 2007) and their cognitive processes can constrict to focus narrowly on unthreatening issues through the avoidance of self-focused attention (Baumeister et al., 2005). Apparently, the immediate response too many meaning-threatening experiences is a defensive, emotional shutdown, which is why emotional distress fails to mediate these behavioral consequences (Twenge et al., 2003). Further, it has been argued that negative affect may later run parallel to these experiences instead of mediating them (De Wall et al., 2010). In sum, recent research findings on consequences of meaning threats are consistent in the conclusion that negative affect does not explain the increase in escape from self-awareness.

A limitation of Study 1’s diary design was its short time period. It would be valuable to measure boredom and eating behaviors over a longer period, accounting for how detailed daily assessment may influence participants’ normal eating habits (O’Connor et al., 2008). Future replications may wish to obtain physiological data for concurrent validity. This would also facilitate the study of some possible moderators such as hunger. In addition, Study 1’s methodology to assess food intake would benefit from using methods that do not entirely rely on self-report (e.g., observational studies). This would provide a better understanding of exactly how many more calories are consumed in response to boredom. Finally, Study 1’s sample mainly consisted of female participants. Yet, there was better gender balance in the following studies suggesting that the phenomenon is generalizable. Nevertheless, we acknowledge that a number of health-related variables could moderate these findings, as discussed later (e.g., dieting status; Goldenberg et al., 2005).

It could be suggested that Study 1’s findings could be interpreted alternatively. That is, participants were primed of Study 1’s purpose and adjusted their eating behaviors accordingly. Likewise, bored people may have better memories for what they consumed during as they were involved in less engaging activities. However, we believe that sufficient empirical evidence exists in Studies 2 and 3 to indicate that the causal direction is likely from experience of boredom to the consumption of foods high in carbohydrates, fats, and sugars and not vice versa. Further, these studies did not rely on recalled behaviors. Numerous experimental studies demonstrate that meaning threats are associated with unpleasant self-awareness and engaging in hedonic behaviors that avoid this focus (e.g., Arndt et al., 1998). Physiological data from other studies lends convergent validity to these claims (e.g., Newman et al., 2007). Further, diary studies have often been shown to be reliable and valid measures of eating behaviors (Bingham et al., 1995, 1997; O’Connor et al., 2008).

There are also several lines of research supporting the association between negative affective states and food intake (Adriaanse et al., 2011). In addition, recall biases generally effects trait measures of eating behaviors rather than state measures, as investigated in this research. Ready et al. (2007) further note that it has been shown that people can report recent affect with moderate accuracy. A literature review also failed to identify any research that suggests a plausible psychological or biological mechanism that indicates that food consumption recall influences emotional perceptions of one’s day (O’Connor et al., 2008).

The findings from our studies provide and interesting foundation for future research in this field. Escaping the self through unhealthy eating may only occur in specific circumstances such as when one is highly self-aware. Future research could further investigate how people low in objective self-awareness deal with the problems associated with boredom. Some health-facilitating experiences may also be threat-avoidance responses (e.g., flow; Csikszentmihalyi, 1975/2000). Additionally, coping variables, more common in certain genders, may moderate health decisions (Arndt et al., 2006). Indeed, longitudinal replications will help to verify under what circumstances bored people may choose different strategies to escape from the self.

In addition, it is possible that different strategies are chosen when different means of meaning-regulation are available simultaneously. Wisman (2006) postulates that those with stronger, coherent worldviews or leanings may attempt to manage meaning-threats with worldview defense (Dechesne et al., 2003; Van Tilburg and Igou, 2011). Those with weaker, less coherent worldviews or those that feel incompetent to live up the standards set by certain cultural norms may be left without adequate resources to regulate meaning symbolically. Therefore, the latter groups (e.g., individuals with low self-esteem) may be more likely to attempt avoiding self-awareness through eating. Thus, unhealthy eating or more generally unhealthy behaviors in response to boredom may occur particularly when individuals experience that symbolic resources are unavailable or insufficient (Wisman, 2006).

## Conclusion

The current findings support our hypothesis about how and why eating may emerge from common experiences like boredom (Koball et al., 2012). This hypothesis can be further examined by future studies that address some of the points raised in our Discussion. Unhealthy behavior may draw attention away from the threatening, self-focused, existential experience that boredom entails. Our results further demonstrated that boredom can promote eating healthy, more exciting foods by endorsing bored people’s need for sensation-seeking. This fascinating finding has great potential in dietary intervention designs (World Health Organization-Europe, 2007; Adriaanse et al., 2009).

## Conflict of Interest Statement

The authors declare that the research was conducted in the absence of any commercial or financial relationships that could be construed as a potential conflict of interest.
